# Lung Deposition of the Dry Powder Fixed Combination Beclometasone Dipropionate Plus Formoterol Fumarate Using NEXThaler^®^ Device in Healthy Subjects, Asthmatic Patients, and COPD Patients

**DOI:** 10.1089/jamp.2016.1359

**Published:** 2018-09-26

**Authors:** Johann Christian Virchow, Gianluigi Poli, Christiane Herpich, Claudius Kietzig, Hilke Ehlich, Daniela Braeutigam, Knut Sommerer, Sabine Häussermann, Fabrizia Mariotti

**Affiliations:** ^1^ Department of Pneumology/Intensive Care Medicine, University of Rostock, Rostock, Germany.; ^2^ Global Clinical Development, Chiesi Farmaceutici S.p.A, Parma, Italy.; ^3^ Inamed GmbH, Gauting, Germany.

**Keywords:** asthma, BDP/formoterol combination, COPD, inhaled drug, lung deposition, NEXThaler^®^ DPI

## Abstract

***Background:*** This study evaluated the lung deposition and the distribution pattern in the airways of a fixed combination of beclometasone dipropionate (BDP) and formoterol fumarate (FF) (100/6 μg) delivered as an extrafine dry powder formulation (mass median aerodynamic diameter, MMAD (μm) BDP = 1.5; FF = 1.4) through the NEXThaler^®^ device in healthy subjects, asthmatics, and patients with COPD.

***Methods:*** Healthy subjects (*n* = 10), asthmatic patients (*n* = 9; 30%≤FEV_1_ < 80%), and COPD patients (*n* = 9; FEV_1_/FVC ≤70%, 30%≤FEV_1_ < 50%) completed this open-label, single administration (inhalation of four actuations) parallel group study. After inhalation of ^99m^Tc-radiolabeled BDP/FF combination (radiolabeled BDP + unlabeled FF), the drug deposition was assessed using a gamma-scintigraphy technique. Patients' lung function was assessed.

***Results:*** No significant difference in drug deposition was observed between the three study groups. Mean lung deposition, extrathoracic deposition, and amount exhaled ranged, respectively, between 54.9% and 56.2%, between 41.8% and 43.2%, and between 1.6% and 3.3% of BDP emitted dose (71.7 ± 2.5 μg) for the three study groups. The central to peripheral ratio (reflecting the lung distribution pattern) ranged between 1.23 and 2.02 for the three study groups, indicating a distribution of the drug throughout the airways, including periphery. The study treatment produced a forced expiratory volume in one second (FEV_1_) increase over time, reaching a maximum improvement generally within 1–4 hours.

***Conclusions:*** The fixed extrafine dry powder combination BDP/FF (100/6 μg) administered through the DPI NEXThaler^®^ achieved similar intrapulmonary deposition in healthy subjects, in asthmatic patients, and COPD patients (approximately 55% of emitted dose) irrespective of the underlying lung disease with a negligible amount of exhaled particles. The study showed high reliability of the device, reproducible dosing, and distribution throughout the lungs. The results supported the concept of efficient delivery of the combination to the target pulmonary regions, thanks to the extrafine formulation. FEV_1_ profile confirmed a relevant pharmacodynamic effect of the product.

## Introduction

Inhaled corticosteroids (ICs) and long-acting beta-2-agonist (LABA) fixed combinations should be considered in asthma and COPD therapy.^(1–6)^

Inhalation devices for aerosol therapy are conventionally divided into three broad categories: Pressurized Metered Dose Inhalers (pMDIs), Dry Powder Inhalers (DPIs), and nebulizers. The pMDI is the most commonly prescribed inhalation system.^(7)^ Despite their convenience and manageability, pMDIs may present some drawbacks for patients who cannot well coordinate or have a preference for DPI systems. For these patients, DPIs may represent a valid alternative to pMDIs, being a delivery system requiring no hand-breath coordination for an efficient inhalation.

Chiesi Farmaceutici S.p.A has developed a DPI (NEXThaler^®^) which is a pocket size, breath actuated multidose reservoir system with an inspiratory flow resistance of 0.036 kPa^1/2^/(L/min) corresponding to a flow rate of 55 L/min at 4 kPa. It is an efficient and easy to use device.^(8)^

NEXThaler^®^ is available in the market for the delivery of an extrafine fixed combination of beclometasone dipropionate (BDP) and formoterol fumarate (FF), which is also available as a pMDI formulation with the name of Foster^®^.^(9–15)^

The objective of this study was to evaluate the lung deposition and the distribution pattern in the airways of the radiolabeled extrafine fixed combination of BDP and FF delivered through NEXThaler^®^ DPI in healthy subjects, asthmatic patients, and COPD patients. The amount of drug deposited in the oropharynx and the influence of airway obstruction on lung deposition were also investigated. The extrathoracic deposition and the exhaled amount of drug, as well as the lung function, were evaluated.

## Materials and Methods

### Study design

This was an open-labeled, uncontrolled, nonrandomized single administration study, consisting of one single treatment of four actuations of ^99m^Technetium (^99m^Tc) radiolabeled BDP/FF fixed combination, delivered by the NEXThaler^®^ device (Chiesi Farmaceutici S.p.A., Italy) yielding a total nominal dose of 400 μg BDP and 24 μg FF, corresponding to the daily dose of the drug.

The primary end point was intrapulmonary deposition of BDP/FF (% of emitted dose). Secondary end points included extrathoracic deposition, amount of BDP/FF exhaled, central to peripheral (C/P) deposition ratio as an index of regional lung deposition, and variance of pixel counts (VAR) as an index of homogeneity of deposition within the lung. The drug deposition was also expressed relative to nominal dose. An exploratory analysis of regional lung deposition was done calculating the central to total (C/T) deposition ratio, the intermediate to total (I/T) deposition ratio, and the peripheral to total (P/T) deposition ratio.

Lung function parameters were also assessed to detect the occurrence of bronchodilation in relation with the lung deposition of the drug. Safety was assessed by documenting all adverse events that occurred during the trial.

Following the screening visit, eligible healthy subjects and patients entered the study. On the day of treatment, subjects inhaled the study medication and remained at the study site for 10 hours to complete the study assessments. The day after, subjects came back to the study site for the 24-hour postdose assessments.

At screening and on administration day at predose, subjects were trained on the correct use of the inhaler. To obtain the optimum aerosol dispersion threshold with the NEXThaler^®^, a Peak Inspiratory Flow (PIF) of at least 40 L/min was required to ensure the activation of the NEXThaler^®^ device and optimum aerosol dispersion. Time 0 for each dose was defined as the time when the inhaler was first actuated. No food was allowed from 3 hours predose until at least 3 hours postdose. Fluid intake was forbidden from 1 hour predose until 1 hour postdose.

The study was conducted in accordance with the Declaration of Helsinki, the ICH Harmonized Tripartite Guideline for Good Clinical Practice, and relevant regulatory requirements. The Ethics Committee of the “Bayerische Landesärztekammer” (Munich, Germany) and the responsible regulatory authorities approved the study protocol.

### Treatment

Each subject inhaled four actuations of radiolabeled 100 μg BDP +6 μg FF (up to 2 MBq per actuation), yielding a total dose of 400 μg BDP and 24 μg FF. The total amount of radioactivity administered was no more than 8 MBq (i.e., four actuations). The total radiation exposure was approximately 96 μSv, while the lung exposure was 65 μSv. The ^99m^Tc-radiolabeled BDP/FF combination (containing radiolabeled BDP + unlabeled FF) was administered using NEXThaler^®^.

### Subject selection

Both male and female subjects, able to properly use the DPI, were recruited. Healthy and asthmatic subjects were aged 21–65 years, and both were required to be nonsmokers or ex-smokers for at least 1 year (previous smoking history of <5 pack years).

Asthmatic patients were required to have moderate persistent or severe persistent asthma,^(4)^ forced expiratory volume in one second (FEV_1_) between 30% and 80% of predicted before bronchodilation, and a FEV_1_ reversibility ≥12% and at least 200 mL of the initial value 15 minutes after inhalation of 200 μg Salbutamol.

COPD patients were aged 40–70 years, with a smoking history of a minimum of 10 pack years. COPD patients were required to have post bronchodilator FEV_1_ between 30% and 50% of predicted values and post bronchodilator FEV_1_/FVC ≤0.70 (absolute value).

All subjects gave written informed consent. Subjects were excluded from the study if they had clinically relevant abnormal laboratory values, and clinically significant and uncontrolled cardiac, hepatic, renal, gastrointestinal, endocrine, metabolic, neurologic, or psychiatric disorders. Patients were not included if they changed the dose or type of asthma/COPD medication within 4 weeks before the screening visit, and if they had experienced an exacerbation in the last 4 weeks.

No LABA, long-acting anticholinergic drugs, theophylline, or BDP were allowed 72 hours before inhalation of study medication. In addition, no β-blockers in the previous 24 hours and no inhaled steroids (with the exception of BDP) in the previous 12 hours were permitted. No short-acting anticholinergic or *β_2_*-agonist drugs were permitted within 8 hours before inhalation of test medication.

### Radiolabeling technique

Radiolabeling was performed at the Inamed laboratories in Gauting considering Good Manufacturing Practice (GMP). ^99m^Tc-Pertechnetate which is dissolved in a 0.9% sodium chloride solution was desalted as described previously^(7)^ and dissolved into a water solution. The ^99m^Tc-water solution was added to the BDP powder, and water was finally evaporated from the suspension under nitrogen stream of 30 L/min in a rotary evaporator.

The radioactively labeled BDP powder was blended with a preblend of FF and lactose carrier using a TURBULA mixer (WAB, Muttenz, Switzerland) and a specific sieving regime to produce the final labeled powder. The resulting bulk powder (with a mean radioactivity between 0.525 MBq and 1.1 MBq per 10 mg bulk powder), was filled into empty NEXThaler^®^ devices.

### Radiolabel validation

The procedure of radioactive labeling of the study medication was validated before the beginning of the study by comparing the radiolabeled product and the unlabeled product (reference product), in terms of particle size distribution and emitted dose, to ensure that the deposition of radioactivity in the respiratory tract after inhalation of the labeled powder represents the deposition of the active components.

The validation of the radiolabeling procedure was performed with three different batches of radiolabeled product and compared to one batch of unlabeled drug product (reference product). The batch content uniformity of the radioactive bulk powder blend was analyzed in terms of radioactivity using a scintillation counter (MiE, Dresden, Germany) and in terms of BDP and formoterol content using a high-performance liquid chromatography (HPLC) system with UV detection (Dionex, Idstein, Germany).

In addition the radiolabeled powder blend was analyzed for degradation products, microbiological contamination, and residual solvents.

NEXThaler^®^ devices containing the radiolabeled product were analyzed in terms of emitted dose and particle size distribution. The emitted dose of both radioactivity and active drugs was evaluated using a Dose Uniformity Sampling Apparatus (DUSA, Copley Scientific, Nottingham, United Kingdom) at a pressure drop across the device of 4 kPa. DUSA was rinsed with solvent.

Radioactivity of rinsing solution was measured in the scintillation counter, while active drugs were analyzed by HPLC.

The emitted dose was tested on three inhalers for each labeled batch (three inhalers were tested once for each batch) and on three inhalers of the unlabeled batch (each inhaler was tested twice).

The particle size distribution was evaluated using an eight-stage Andersen Cascade Impactor at the same flow rate as used for the emitted dose (56–59 L/min). For the assessment of particle size distribution, one inhaler of each radiolabeled batch was tested once, while three inhalers of the unlabeled batch were tested twice. After firing five actuations of the radiolabeled formulation into the impactor, the radioactivity on each impactor stage and on the impactor throat, on preseparator and on the exhalation filter was measured, then all plates were washed, and the BDP and formoterol amount on each stage were analyzed.

The mass median aerodynamic diameter (MMAD), fine particle dose (FPD; particles <4.7 μm), and fine particle fraction (FPF) were determined by CITDAS evaluation software version 2.0 (Copley Scientific Ltd, United Kingdom) using a probit analysis.

### Batch release of ^99m^Tc-radiolabeled BDP/FF combination

On each study day, before administration, the powder batch was analyzed to assess radioactivity content uniformity, and each filled NEXThaler^®^ device was analyzed to determine the emitted dose of radioactivity per actuation. In one of the filled NEXThaler^®^ devices the particle size distribution was analyzed by radioactivity.

The results of these analyses were used for releasing the batch. Release criteria were radioactivity content uniformity of the powder batch of 0.525–2.1 MBq per 10 mg powder (CV ≤5%), radioactivity of the emitted dose from the device of 0.5–2.0 MBq per actuation, and radioactivity of the FPF of >35% of the mean radioactive content.

### Test drug inhalation

Before test drug inhalation, subjects were trained to use the NEXThaler^®^ properly using a placebo device. To control and to standardize the inhalation flow, an In-Check DIAL device (Clement Clarke International, Essex, United Kingdom) simulating the internal resistance of the NEXThaler^®^ device was used. Subjects were instructed to inhale with an inspiratory flow of at least 40 L/min to ensure the activation of the NEXThaler^®^ device and optimum aerosol dispersion.

Subjects inhaled four actuations of BDP/FF (100/6 μg) using the breathing pattern they had learned with the placebo inhaler and the In-Check DIAL device. At the end of the inspiration, subjects were asked to breath-hold for 5 seconds and then to exhale into an exhalation filter. Total duration of inhalation procedure was 2–2.5 minutes. No more than 8 MBq ^99m^TC-BDP was administered to each subject during the study.

### Measurement of lung deposition

Immediately after dosing, a gamma camera image was taken (sequence of four one minute images) using a MIE LFOV Digitrac 66 gamma camera with a field of view of 61 × 39 cm and a low energy parallel whole collimator. An ^81m^Krypton-ventilation scan was also obtained to define the lung borders and lung fields.

Subjects were sitting upright with their back to the gamma camera, and images were taken posterior only. Using the regions of interest (ROIs) defined from this ventilation scan, the following parameters were obtained from the first gamma camera image of the abovementioned sequence:

- The count rates measured for the lung region (C_L_)
- The count rates measured for the extrathoracic region, including oropharynx, trachea, esophagus, and stomach, corrected for tissue attenuation (C_E_)

All acquired lung deposition counts were corrected for background radiation, radioactive decay, and tissue attenuation.

The radioactivity on the exhalation filter (A_F,X_) and the radioactivity emitted by the inhaler (A_I_) which was determined during batch release measurements were measured using a high-sensitive scintillation counter.

From these count rates and activity data the following parameters were calculated:

- Absolute activity deposited in the lungs (A_Re, L_) A_Re, L_ = (A_I_-A_F,X_). (C_L_/C_L_ + C_E_)
- Lung deposition relative to emitted dose (D_L,E_): D_L,E_ = A_Re,L_/A_I_
- Absolute activity deposited in the extrathoracic region (A_Re, E_): A_Re.E_ = (A_I_–A_F,X_). (C_E_/C_L_ + C_E_)
- Extrathoracic deposition relative to emitted dose (D_E, E_): D_E, E_ = A_ReE_/A_I_
- Fraction of exhaled activity relative to emitted dose (M_X, E_): M_X, E_ = A_F,X_/A_I_

Tissue attenuation corrections were performed according to Pitcairn.^(16)^

### Estimation of drug deposition relative to the nominal dose

Lung deposition, extrathoracic deposition, and fraction of exhaled drug were also expressed relative to the nominal dose, starting from the deposition relative to emitted dose and from the emitted dose as measured by HPLC detection for the labeled batch (Table 2). Only the emitted dose of BDP was considered in the calculation, since BDP was the labeled compound in the administered powder.

The drug deposition relative to nominal dose (D,_N_) was calculated by:

D,_*N*__=_ (μg deposited/BDP nominal dose) ·100

Where the amount of drug deposited in each compartment was estimated by:

μg deposited = (D_relative to emitted dose_/100) · BDP emitted dose

### C/P ratio

**FIG. 1.**
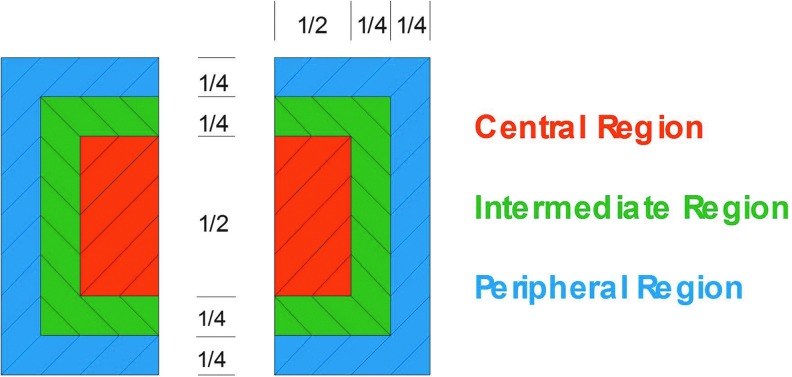
Definition of central, intermediate, and peripheral regions of interest relative to the total lung.

For the determination of C/P ratios of deposited activity after inhalation, whole lung rectangular ROIs for each lung were drawn at the boundaries of the Krypton-ventilation scan. Central ROIs were positioned on the interior boundary of the lung and correspond to 25% of the area of the whole lung. The peripheral region (P) was that area lying between the intermediate region and whole lung outline (Fig. 1).

The measured counts in the central and peripheral ROIs were divided by the counts of the whole lung region.^(7)^ The calculated ratios were then normalized using the ratios calculated for the Krypton-ventilation scan. The parameter C/P represents the mean value of the left and right lung ratios. Decreases in C/P reflect a decrease in large bronchial airway deposition relative to intermediate/small bronchi/bronchioles and alveolated airspaces.

An exploratory analysis was performed describing intermediate (I) and peripheral (P) ROIs, as shown in Figure 1, corresponding to 31% and 44% of the area of the whole lung, respectively, and calculating the central to total deposition (C/T) ratio, the intermediate to total deposition (I/T) ratio, and the peripheral to total deposition (P/T) ratio.

### Variance of pixels

As an additional analysis for homogeneity of deposition, the variance of pixel counts (VAR) in the lung was calculated from the pixel count histogram (pixel counts vs. total counts) of the left and right lung ROIs of the scintigraphy lung images. The parameter VAR represents the mean value of the left and right variance.^(7)^ As homogeneity of deposition within the lung improves, the VAR decreases.

### Measurements of lung function

Spirometric lung function parameter FEV_1_ was measured using a Jaeger-Masterlab and Master-Scope (Cardinal Health, Würzburg, FRG) at screening (pre and post salbutamol during the reversibility test) and on administration day predose, 15, 30 minutes, 1, 2, 4, 6, 8, 10, and 24 hours postdose to fully monitor the lung function of subjects after the intake of BDP/FF.

It has to be noted that in this study a single administration of a dose corresponding to the therapeutic daily dose of the product was administered by four inhalations, while in the clinical practice the same dose is administered by two inhalations twice daily.

Three technically satisfactory measurements were done for each patient and the highest value recorded. Predicted values were calculated according to the Quanjer formula's of the European Coal and Steel Community.^(17)^

### Statistical and analytical plans

Variables were compared between subject groups by an analysis of variance (ANOVA) using a linear model with subject group as independent variable. The model was calculated assuming group as fixed effect using the SAS procedure “Mixed”. Correlations between baseline lung function and deposition parameters were tested using Spearman rank correlation analysis.

Sample size of eight subjects to detect differences in lung deposition between groups of about 30% (paired *t*-test) was roughly estimated on the basis of previous deposition studies where a similar sample size showed reliable and consistent lung deposition results.^(7,18,19)^

## Results

### Subjects

A total of 30 subjects were recruited into the study: 12 healthy subjects, 9 asthmatic patients, and 9 COPD patients. Twenty-eight subjects completed the study: 10 healthy subjects, 9 asthmatic patients, and 9 COPD patients. Two healthy subjects discontinued before receiving any study medication.

Healthy subjects were younger (27.5 ± 9.3 years) than asthmatic patients (49.1 ± 11.6 years) and COPD patients (61.8 ± 5.9 years). The overall gender distribution in the study was balanced [males: *n* = 15 (53.6%), females: *n* = 13 (46.4%)]. All subjects were Caucasian. Baseline data are presented in Table 1.

**Table 1.** Subjects' Baseline Characteristics

	*Group*		
*Variable*	*Healthy subjects, (*n* = 10)*	*Asthma patients, (*n* = 9)*	*COPD patients, (*n* = 9)*
Age (years)	27.5 ± 9.25	49.11 ± 11.56	61.78 ± 5.91
Height (cm)	176.0 ± 10.99	168.33 ± 5.41	170.44 ± 8.99
Weight (Kg)	74.1 ± 16.56	66.88 ± 8.83	74.72 ± 12.99
PIF^a^ (L/min)	76.4 ± 10.5	67.3 ± 14.6	69.9 ± 8.0
FEV_1_ (L)	4.01 ± 0.88	2.08 ± 0.44	1.12 ± 0.34
FEV_1_ (% predicted)	101.5 ± 9.07	72.78 ± 7.6	37.22 ± 7.66
FVC (L)	4.94 ± 1.08	3.33 ± 0.57	2.77 ± 0.64
MEF_25_ (L/s)	1.79 ± 0.76	0.44 ± 0.29	0.17 ± 0.05
MEF_50_ (L/s)	4.38 ± 1.19	1.39 ± 0.60	0.4 ± 0.16
MEF_75_ (L/s)	7.31 ± 1.53	2.81 ± 1.12	0.95 ± 0.38

Results are expressed as mean ± standard deviation.

^a^ Value measured during subjects' training immediately before dosing.

PIF, peak inspiratory flow; FEV_1_, forced expiratory volume in one second; MEF_25_, maximal expiratory flow at 25% vital capacity; MEF_50_, maximal expiratory flow at 50% vital capacity; MEF_75_, maximal expiratory flow at 75% vital capacity; FVC, forced vital capacity.

### Validation of radiolabeling procedure

Three different batches of radiolabeled product were compared to one batch of unlabeled reference product. The labeled BDP/FF NEXThaler^®^ DPI showed minimal differences compared to the reference product.

**FIG. 2.**
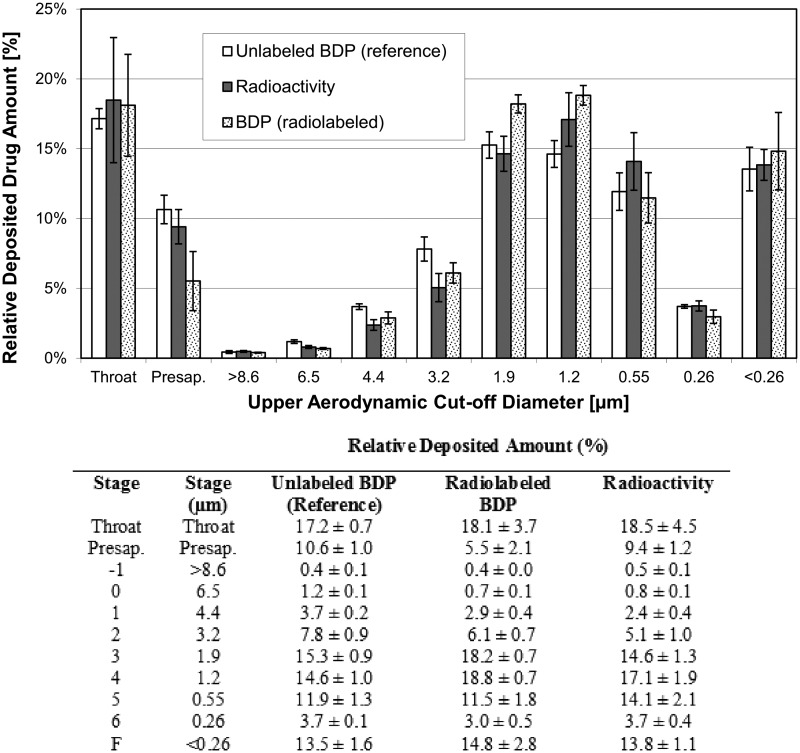
Particle size distribution of unlabeled BDP (reference) and radiolabeled BDP. Radiolabeled BDP measured by radioactivity and by ACI and HPLC (mean ± SD of three batches). Unlabeled BDP (reference) measured by ACI and HPLC only (mean ± SD of three inhalers from one batch). ACI, Andersen Cascade Impactor; BDP, beclometasone dipropionate; HPLC, high-performance liquid chromatography.

**FIG. 3.**
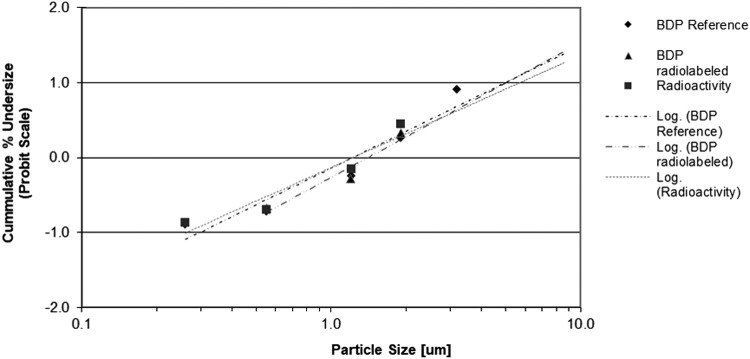
Log-probability plot of cumulative particle size distribution of unlabeled BDP (reference) and radiolabeled BDP. Radiolabeled BDP measured by radioactivity and by ACI and HPLC (mean of three batches). Unlabeled BDP (reference) measured by ACI and HPLC only (mean of three inhalers from one batch).

**FIG. 4.**
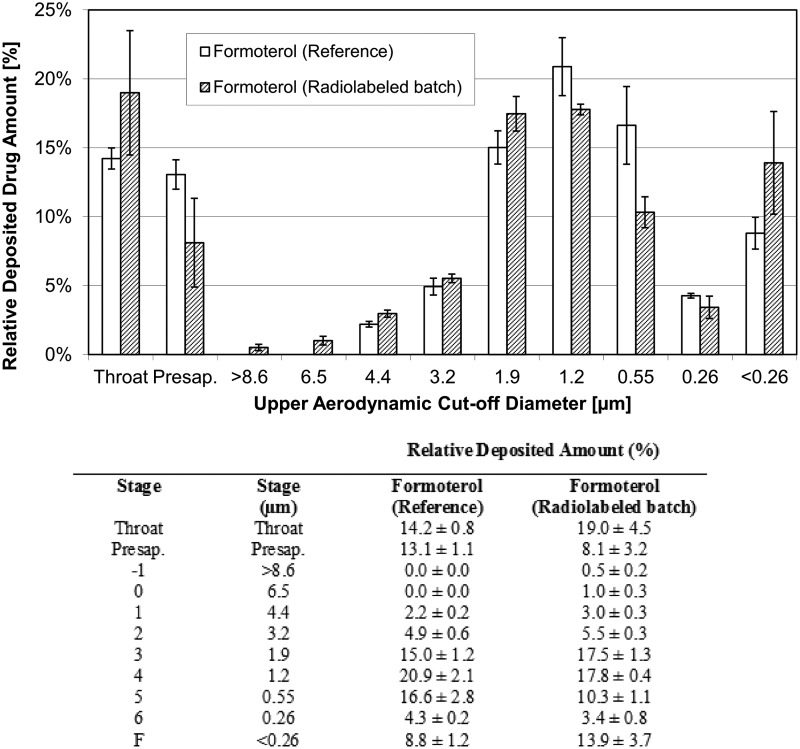
Particle size distribution of Formoterol in the formulation containing unlabeled BDP (reference) and in the formulation containing radiolabeled BDP. Formoterol measured by ACI and HPLC in both formulations (mean ± SD of three radiolabeled batches and of three inhalers from one unlabeled batch).

Particle size distribution of the labeled product measured by radioactivity and by HPLC determination of BDP and FF closely matched the particle size distribution of the reference product (Figs. 2–4 and Table 2).

The emitted dose (ED) (mean ± standard deviation) of FF was 4.4 ± 0.2 μg (*n* = 6) and 4.9 ± 0.3 μg (*n* = 9) for the unlabeled and labeled drug, respectively. Similarly, the ED of BDP was 73.3 ± 3.8 μg (*n* = 6) and 71.7 ± 2.5 μg (*n* = 9) for the unlabeled and labeled drug, respectively.

**Table 2.** Mean and Standard Deviation of Mass Median Aerodynamic Diameter, Emitted Dose, Fine Particle Dose, and Fine Particle Fraction as Determined During Radiolabeling Validation

	*MMAD*^a^*(μm)*	*FPD*^b^*(μg)*	*FPF*^b^*(% of emitted dose)*	*ED*^c^^*^μg;^#^kBq
BDP, unlabeled batch (HPLC detection)	1.53 ± 0.05	56.4 ± 3.8	68.1 ± 1.9	73.3 ± 3.8^*^
Formoterol, unlabeled batch (HPLC detection)	1.43 ± 0.05	3.3 ± 0.3	71.2 ± 1.9	4.4 ± 0.2^*^
BDP, labeled batch (HPLC detection)	1.50 ± 0.00	66.0 ± 3.8	74.1 ± 1.3	71.7 ± 2.5^*^
Formoterol, labeled batch (HPLC detection)	1.53 ± 0.06	3.7 ± 0.12	69.6 ± 2.0	4.9 ± 0.3^*^
BDP + Formoterol, labeled batch (radioactivity detection)	1.40 ± 0.10	—	69.4 ± 3.0	943 ± 93^#^

^a^ MMAD: for each of the three labeled batches, one inhaler was tested once (*n* = 3); three inhalers of one unlabeled batch were tested twice (*n* = 6).

^b^ FPD and FPF: for each of the three labeled batches, one inhaler was tested once (*n* = 3); three inhalers of one unlabeled batch were tested twice (*n* = 6).

^c^ ED: for each of the three labeled batches, three inhalers were tested once (*n* = 9); three inhalers of one unlabeled batch were tested twice (*n* = 6).

ED, emitted dose; FPD, fine particle dose; FPF, fine particle fraction; HPLC, high-performance liquid chromatography; MMAD, mass median aerodynamic diameter.

^*^ represents data expressed as μg; ^#^represents data expressed as kBq.

The FPF (expressed as% of the ED) (mean ± standard deviation) of FF was 71.2% ± 1.9% (*n* = 6) and 69.6% ± 2.0% (*n* = 3) for the unlabeled and labeled drug, respectively. Consistently, the FPF of BDP was 68.1% ± 1.9% (*n* = 6) and 74.1% ± 1.3% (*n* = 3) for the unlabeled and labeled drug, respectively. The MMAD was 1.43 ± 0.05 μm (*n* = 6) and 1.53 ± 0.06 μm (*n* = 3) for formoterol in the unlabeled and labeled product, respectively, and 1.53 ± 0.05 μm (*n* = 6) and 1.50 ± 0.00 μm (*n* = 3) for BDP in the unlabeled and labeled product, respectively.

These results confirm that the labeling procedure did not change the properties of the extrafine unlabeled product. In particular, the similarity of FPF (representative of the respirable fraction of the drug) and MMAD between unlabeled and labeled drug suggests no impact of the labeling process on the aerodynamic behavior *in vivo* of the drug.

### Batch release of ^99m^Tc-radiolabeled BDP/FF combination before administration

At each time of inhalation, a powder batch was prepared, and six or seven NEXThaler^®^ devices were filled. Six powder batches were prepared in total during the study. Every batch was tested before administration. Release criteria were radioactivity content uniformity of the powder batch of 0.525–2.1 MBq per 10 mg powder (CV ≤5%), radioactivity of the emitted dose measured for each filled device of 0.5–2.0 MBq per actuation, and radioactivity of the FPF of >35% of the mean radioactive content.

All the powder batches and the filled NEXThaler^®^ devices used for subjects' treatment fulfilled the release criteria, showing radioactivity content of the powder batch ranging from 0.631 to 1.55 MBq per 10 mg powder (CV from 1.0% to 4.2%), radioactivity of the emitted dose ranging from 0.5 to 1.2 MBq per actuation, and radioactivity of the FPF ranging from 50% to 71% of the mean radioactive content.

### Deposition data

Drug deposition was expressed as percent of the emitted dose of BDP (71.7 ± 2.5 μg as calculated during the validation of the radio labeling process, Table 2) and was comparable in healthy subjects and patients.

The intrapulmonary deposition (D_L,E_) (mean ± standard deviation) accounted for 55.2% ± 3.7%, 56.2% ± 5.8%, and 54.9% ± 4.9% of the emitted dose in healthy subjects, in asthmatic patients and in COPD patients, respectively. The extrathoracic deposition (D_E, E_) (mean ± standard deviation) accounted for 43.1% ± 4.2%, 41.8% ± 5.6%, and 41.8% ± 4.8% of the emitted dose in healthy subjects, in asthmatic patients, and in COPD patients, respectively.

**FIG. 5.**
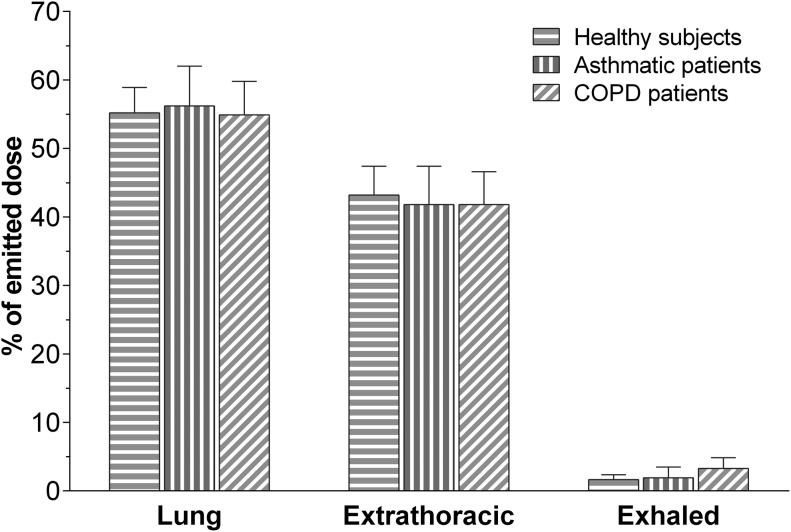
Histogram showing mean (±SD) drug deposition in healthy subjects (*n* = 10), asthma patients (*n* = 9), and COPD patients (*n* = 9), after inhalation of four actuations of BDP/FF (100/6 μg) NEXThaler^®^. FF, formoterol fumarate.

The absolute differences of intrapulmonary and extrathoracic deposition between healthy subjects, asthmatic patients, and COPD patients were negligible, and the analysis of variance (ANOVA) did not show any differences (Table 3 and Fig. 5). The amount of exhaled drug did not differ significantly between groups, being the exhaled fraction (M_X,E_) (mean ± standard deviation) 1.6% ± 0.8%, 1.9% ± 1.6%, and 3.3% ± 1.6% of the emitted dose in healthy subjects, in asthmatic patients, and in COPD patients, respectively (Table 3 and Fig. 5).

**Table 3.** Deposition in Healthy Subjects and Asthmatic and COPD Patients Following Inhalation of Four Actuations of the BDP/FF (100/6 μg) NEXThaler^®^ Radiolabeled Formulation

	*Group*		
*Variable*	*Healthy subjects,* n* = 10*	*Asthma patients,* n* = 9*	*COPD patients,* n* = 9*
Lung deposition (% emitted dose)	55.2 ± 3.7 (50.5; 62)	56.2 ± 5.8 (45.3; 63.6)	54.9 ± 4.9 (48.5; 64.2)
Extrathoracic deposition (% emitted dose)	43.2 ± 4.2 (35.7; 48.8)	41.8 ± 5.6 (34.5; 53.7)	41.8 ± 4.8 (33.8; 46.9)
Amount exhaled (% emitted dose)	1.63 ± 0.72 (0.80; 3.00)	1.92 ± 1.55 (0.40; 5.80)	3.28 ± 1.56^*^ (1.30; 5.40)
C/P	1.23 ± 0.19 (0.95; 1.54)	2.02 ± 0.59^**^ (1.41; 3.27)	1.57 ± 0.29 (1.29; 2.03)
C/T	1.12 ± 0.11 (0.94; 1.27)	1.47 ± 0.19 (1.25; 1.80)	1.29 ± 0.13 (1.16; 1.48)
P/T	1.18 ± 0.06 (1.11; 1.29)	0.96 ± 0.13 (0.77; 1.11)	1.07 ± 0.07 (0.92; 1.17)
VAR (pixel counts)	0.0001 ± 0.0000 (0.0001; 0.0001)	0.0003 ± 0.0002^***^ (0.0001; 0.0008)	0.00018 ± 0.00007 (0.0001; 0.0003)

Results are presented as mean ± standard deviation (range).

^*^ *p* = 0.0116 versus healthy subjects.

^**^ *p* = 0.0002 versus healthy subjects.

^***^ *p* = 0.0029 versus healthy subjects.

Lung deposition expressed relative to the nominal dose was 39.6% ± 2.6% in healthy subjects, 40.3% ± 4.1% in patients with asthma, and 39.4% ± 3.5% in patients with COPD, while the extrathoracic deposition was 30.9% ± 3.0%, 30.0% ± 4.0%, and 30.0% ± 3.4% in healthy subjects, asthmatic patients, and COPD patients, respectively. The amount exhaled expressed relative to the nominal dose ranged approximately between 1.2% and 2.4% for the three groups.

**FIG. 6.**
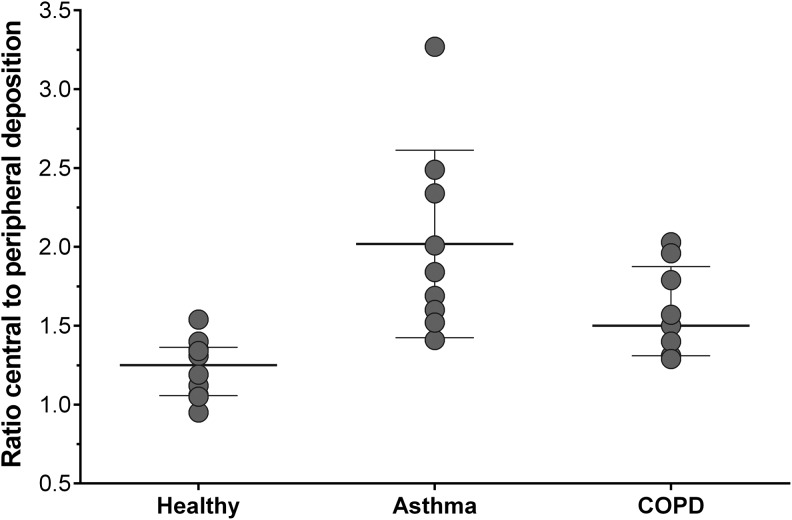
Central to peripheral deposition (C/P) in healthy subjects (*n* = 10), asthmatic patients (*n* = 9), and COPD patients (*n* = 9) after inhalation of four actuations of BDP/FF (100/6 μg) NEXThaler^®^.

The distribution pattern in the lung, evaluated by measuring the central to peripheral deposition (C/P) ratio, confirmed a drug distribution throughout the entire bronchial tree in all three subject groups. However, the mean C/P ratio was significantly (*p* = 0.0002) higher in asthmatics (2.02 ± 0.59) compared to healthy subjects (1.23 ± 0.19), while the difference was less evident for COPD patients (1.59 ± 0.29; *p* = 0.0661) (Table 3 and Fig. 6).

C/P data were in agreement with the results of the exploratory analysis measuring the central to total deposition (C/T 1.12 ± 0.11, 1.47 ± 0.19, and 1.29 ± 0.13 in healthy subjects, asthmatic patients, and COPD patients, respectively) ratio, the intermediate to total deposition (I/T 0.96 ± 0.03, 0.82 ± 0.08, and 0.89 ± 0.06 in healthy subjects, asthmatic patients, and COPD patients, respectively) ratio, and the peripheral to total deposition (P/T 1.18 ± 0.06, 0.96 ± 0.13, and 1.07 ± 0.07 in healthy subjects, asthmatic patients, and COPD patients, respectively) ratio.

Consistently, VAR indicated a significantly more heterogeneous deposition in the lungs of asthmatic patients (0.3 × 10^−3^ ± 0.22 × 10^−3^) compared with healthy subjects (0.1 × 10^−3^ ± 0.0) (*p* = 0.0029) (Table 3) and showed a significant correlation with baseline FEV1 (Spearman correlation coefficient 0.70, *p* = 0.0343). The VAR in COPD patients (0.18 × 10^–3^ ± 0.07 × 10^–3^) did not differ significantly from healthy subjects.

**FIG. 7.**
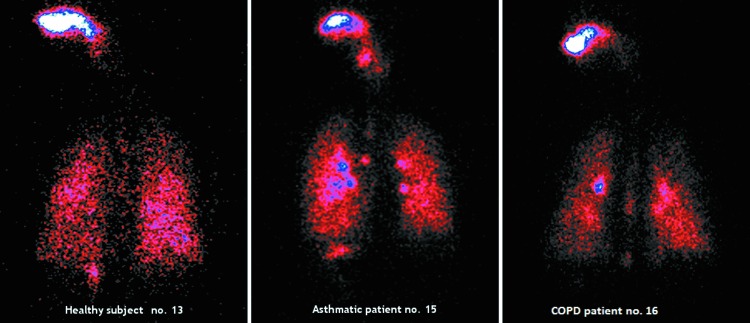
Scintigraphy in individual subject (healthy subject no. 13: D_L_ 55.2%, D_E_ 42.2%, C/P 1.12; asthmatic patient no. 15: D_L_ 57.4%, D_E_ 41.3%, C/P 2.01; COPD patient no. 16: D_L_ 53.6%, D_E_ 42.8%, C/P 1.40%).

**FIG. 8.**
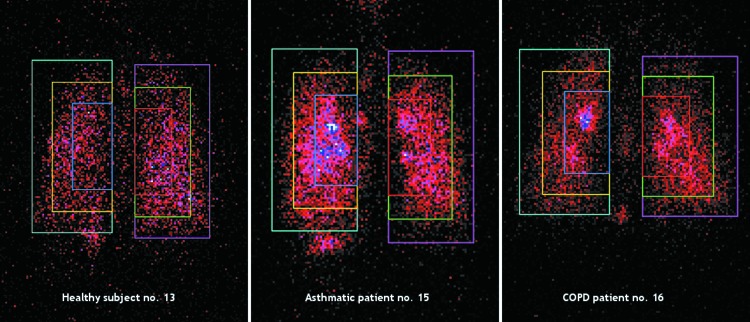
Definition of central, intermediate, and peripheral regions of interest relative to the total lung in individual subject (healthy subject no. 13; asthmatic patient no. 15; COPD patient no. 16).

**FIG. 9.**
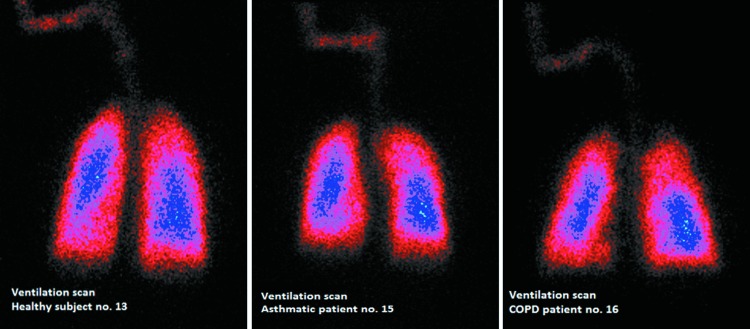
^81m^Krypton-ventilation scan in individual subject (healthy subject no. 13; asthmatic patient no. 15; COPD patient no. 16).

The visual examinations of gamma camera pictures taken in asthmatic patients showed some “hot spots” (dense areas and white or blue colored areas) of drug deposition in the central regions which are known to be related to local bronchial obstruction and to mucus accumulation on the bronchial wall and could be related to the higher C/P ratio and the lower homogeneity observed in asthmatics (see scintigraphy images in Fig. 7, scintigraphy images with definition of ROIs in Fig. 8, and ventilation scan images in Fig. 9).

An outlier C/P ratio value with no apparent explanation was observed for one subject in the asthmatic patients' group. The subject was a female, with Body Mass Index 27.8 kg/m^2^, predose FEV1 1.52 L (62% of predicted), and no deviations reported during the drug inhalation. This subject showed the following deposition parameters: DL,E 62.9%, DE,E 35.5%, and C/P 3.27 and reached a peak FEV1 of 2.06 L (corresponding to 84% of predicted) 30 minutes postdose. The Gamma Camera image of this subject showed unusual hot spots in the lungs, possibly reflecting temporarily airway obstructions due to mucus.

The COPD patients showed fewer hot spots than the asthmatic patients; however, not ventilated regions of the lungs, which had less or no drug deposited, were visible in the gamma camera pictures. “Hot spots” were also visible in the oropharynx, related to the amount of drug deposited in the oral cavity.

Baseline lung function of healthy subjects and asthmatic and COPD patients was markedly different, being predose mean (±standard deviation) FEV_1_ 4.01 ± 0.88 L, 2.08 ± 0.44 L, and 1.12 ± 0.34 L, respectively (corresponding to 101% ± 9%, 73% ± 8%, and 37% ± 8% of the predicted value, respectively), see Table 1.

On the contrary, mean PIF values, as measured by In-Check dial device during subjects' training on administration day, were comparable between groups (76.4, 67.3, and 69.9 L/min in healthy subjects, asthmatic patients, and COPD patients, respectively), see Table 1, suggesting lack of relationship between FEV_1_ and PIF and supporting the ability of patients with significantly compromised lung function to generate sufficient inspiratory flows through medium resistance DPIs.

Consistently, the results of statistical analysis indicate that the correlation between baseline lung function parameters and parameters describing lung deposition was generally moderate to poor, with the exception of VAR for asthmatic patients as reported above.

### Lung function

**FIG. 10.**
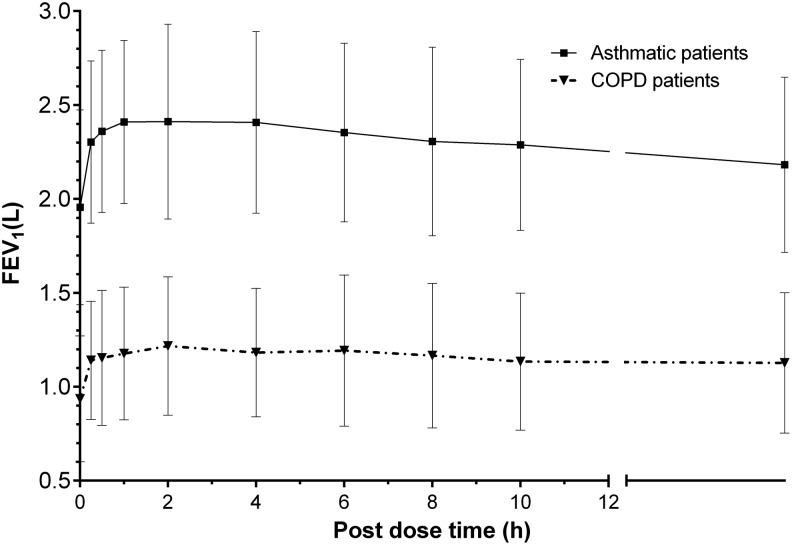
Mean (±SD) forced expiratory volume in 1 second (FEV1) over time in asthmatic patients (*n* = 9) and COPD patients (*n* = 9) after inhalation of four actuations of BDP/FF (100/6 μg) NEXThaler^®^. FEV1, forced expiratory volume in one second.

Administration of the ^99m^Tc-labeled BDP/FF combination using NEXThaler^®^ produced a FEV_1_ increase over time, reaching a maximum improvement generally within 1–4 hours postdose and declining over the next 12–24 hours. The maximum FEV_1_ increase from predose was approximately 450 mL, 1 hour postdose in asthmatic patients, while a lower increase (280 mL, 2 hours postdose) was observed in COPD patients (Fig. 10).

### Safety evaluation

Overall, five adverse events were observed all reported by asthmatic patients. They were of mild (*n* = 3) or moderate (*n* = 2) intensity and were not considered related to the study medication. Mild and moderate headache were reported by one and two patients, respectively. Other adverse events were mild gastrointestinal infection and mild oral herpes.

## Discussion

This study showed that the extrafine fixed combination of BDP/FF delivered through NEXThaler^®^ led to a high lung deposition of the drug (approximately 55% of emitted dose and 40% of nominal dose) irrespectively of subjects' lung disease. The exhaled amount of extrafine particles from NEXThaler^®^ was negligible (ranging from 1.6% to 3.3% of emitted dose) and in the range observed with nonextrafine formulations,^(20)^ indicating that a smaller particle size is not associated with a higher exhaled fraction.

Drug was distributed throughout the lung, as supported by the central to peripheral deposition (C/P) ratio. In the central lung regions of asthmatic patients, gamma camera images evidenced some dense and white or blue colored areas corresponding to “hot spots” of drug deposition which were reflected in slight, although significantly reduced, homogeneity of drug distribution (C/P ratio and VAR) in asthmatic patients compared to healthy subjects.

COPD patients showed fewer hot spots. Not ventilated regions of the lungs with less drug deposited were visible instead, but the impact on homogeneity of drug distribution in COPD patients was not significant.

Dose delivery from DPIs such as NEXThaler^®^ is reliant upon patients being able to generate an adequate PIF to disaggregate the drug into particles small enough to reach the lungs. In the present study, pulmonary disease status had only a limited influence on drug deposition and exhalation patterns, and no apparent relationship was found between baseline FEV_1_ and PIF, as reported also in the literature.^(21)^ These findings indicate that even in patients with significantly compromised lung function, relatively consistent inspiratory flows can be generated through the DPI NEXThaler^®^, as also supported by a previous study, which demonstrated the capability of adult patients with different degrees of asthma control to use effectively the NEXThaler^®^.^(22)^

The DPIs, as mentioned before, have some advantages over the MDIs, in reducing coordination problems in some patients.^(23,24)^ However, the development of DPIs is difficult to achieve,^(25,26)^ and formulations and de-aggregation systems available for marketed DPIs could only deliver drug with a considerably larger particle size than the ones delivered through extrafine HFA(hydrofluoroalkane)-pMDIs.

Deposition patterns of extrafine BDP/FF combination and of ICs and LABAs administered as single agents through pMDI formulations have been investigated in the past.^(7,19,27,28)^ The deposited dose in the lungs after inhalation of extrafine HFA pMDI BDP/FF (Modulite technology) was 34%, 31%, and 33% of the nominal dose in healthy subjects, asthmatic patients, and COPD patients, respectively.^(7)^ Extrafine particles generated through HFA-pMDIs achieved reaching small airways and reducing mouth and throat deposition.^(29)^ NEXThaler^®^ is the only existing DPI releasing extrafine particles associated with an MMAD of approximately 1.5 μm.

Clinically, it is important that the drug reaches the areas in the lungs, where it is most effective. Evidence shows that in asthma and COPD the small airways are a main site of obstruction and, at the same time, the main site of airway inflammation.^(30,31)^

In the present study, the deposition of the drug in all regions of the lungs, including our defined peripheral lung region, regardless of the state of disease was observed. This finding is consistent with other data obtained with extrafine particles.^(7,19,28,32)^

After inhalation of BDP/FF delivered through NEXThaler^®^, the improvement in FEV_1_ observed mainly in asthmatic patients reached its maximum after 1–4 hours and declined over the next 12–24 hours.

It has to be noted that in the present study subjects received four inhalations of the drug in a single administration, while in the clinical practice the dosing regimen consists of two inhalations twice daily. Therefore, the lung function response in the present study cannot be directly compared to data reported in literature at the therapeutic regimen. However, the FEV_1_ profiles confirm the prolonged activity of the BDP/FF combination delivered through NEXThaler^®^.

The treatment was well tolerated. No serious adverse events were reported in any of the study groups. These data are in agreement with data from the literature, which generally state that the safety profile of BDP/formoterol does not differ from that of other available IC/LABA combinations.

In conclusion, in the present study, administering the BDP/FF daily dose in a single administration, NEXThaler^®^ device has shown high reliability and reproducible dosing for healthy subjects, as well as asthmatic and COPD patients in different stages of their disease. The high lung deposition, the negligible exhaled fraction, and the resulting low extrathoracic deposition show an efficient delivery of drug to the target region.

## Notes

Reviewed by:

Steve Newman

Myrna Dolovich

Peter Byron
